# Zinc Concentration in Breast Milk Is Inversely Correlated with the Zinc Supplementation Requirements of Preterm Infants

**DOI:** 10.3390/nu17050840

**Published:** 2025-02-28

**Authors:** Tokuo Miyazawa, Madoka Shirai, Yutaro Noguchi, Kazuna Haruyama, Kosuke Oikawa, Akio Ebata, Tomomasa Terada, Yoshiyuki Hasebe, Katsumi Mizuno

**Affiliations:** Department of Pediatrics, Showa University School of Medicine, 1-5-8, Hatanodai, Shinagawa-ku, Tokyo 142-8666, Japan

**Keywords:** zinc, preterm infants, breast milk, human milk fortification, enteral nutrition

## Abstract

**Background:** Zinc is an essential trace element that is crucial for numerous biological processes, including protein synthesis, antioxidant activity, and bone calcification. Preterm infants are at high risk of zinc deficiency owing to inadequate zinc stores at birth and the rapid decline in zinc concentration in breast milk. This study aimed to evaluate the relationship between zinc concentrations in breast milk and zinc supplementation in preterm infants. **Methods:** A prospective observational study was conducted at Showa University Hospital, enrolling preterm infants born at less than 32 weeks of gestation or with a birth weight of less than 1800 g. Serum zinc levels, breast milk zinc concentrations, and zinc acetate supplementation were analyzed. **Results:** The results indicated an inverse correlation between breast milk zinc concentration and the required zinc supplementation dose. Infants receiving high-dose zinc supplementation (≥3 mg/kg/day) had significantly lower breast milk zinc concentrations at 2, 4, and 5 weeks postpartum. **Conclusions:** These findings highlight the need for individualized zinc monitoring and supplementation strategies to prevent zinc deficiency in preterm infants. Considering the absence of zinc in human milk fortifiers in Japan, aggressive zinc supplementation may be necessary to ensure optimal growth and development.

## 1. Introduction

Zinc is an essential trace element for humans that is critical for protein synthesis, DNA/RNA synthesis, antioxidant function, and bone calcification [1,2,3]. Because the transfer and accumulation of zinc from the mother to the fetus mainly occur in the later stages of pregnancy, preterm infants are born without sufficient zinc stores in their bodies [3,4,5]. Although enteral nutrition with breast milk is considered the basis of nutritional management for preterm infants, the zinc concentration in breast milk rapidly decreases during the transition from colostrum to mature milk; therefore, there is a possibility that preterm infants will suffer from zinc deficiency if they are fed breast milk alone [6,7].

Furthermore, rapid growth after birth has been associated with zinc deficiency in preterm infants [8]. While early aggressive nutrition in extremely preterm infants promotes growth, it may also increase the risk of zinc deficiency [9]. In addition, after weaning from parenteral nutrition, human milk fortification is used, because breast milk alone cannot often provide sufficient nutrition (i.e., protein, energy, calcium, and phosphorus) to ensure appropriate growth in preterm infants. In Japan, the addition of zinc to breast milk substitutes is permitted. However, since human milk fortifiers (HMS-1 and HMS-2, Morinaga Milk Industry, Tokyo, Japan) are not classified as breast milk substitutes, zinc is not added due to legal restrictions [10]. Therefore, oral administration of zinc acetate is commonly used in Japan to prevent and treat zinc deficiency in preterm infants.

The *Practice guideline for zinc deficiency* published by the Japanese Society of Clinical Nutrition recommends a daily dose of 1–3 mg/kg for infants and children with zinc deficiency but does not specify a specific dose for preterm infants [11]. In contrast, in Western guidelines, the recommended zinc dosage for preterm infants is 1.4–2.5 mg/kg/day by the American Academy of Pediatrics (AAP) [12] and 2–3 mg/kg/day by the European Society for Pediatric Gastroenterology, Hepatology and Nutrition (ESPGHAN) [13].

In clinical practice, we sometimes encounter cases where it is challenging to maintain serum zinc levels at the recommended daily dose of 1–3 mg/kg/day. This may be due to individual variations in the zinc concentration of breast milk, which is the sole source of zinc for preterm infants who have been weaned from intravenous nutrition. In Japan, because human milk fortifiers do not contain zinc, preterm infants ingest breast milk with a low zinc content and are at a high risk of zinc deficiency. Therefore, more aggressive zinc supplementation is necessary. In this study, to clarify the effect of zinc concentration in breast milk on zinc deficiency in preterm infants, we evaluated the relationship between the changes in zinc concentration in breast milk and the dose of zinc acetate administered to infants.

## 2. Materials and Methods

This single-center prospective observational study was conducted on preterm and low-birthweight infants admitted to the NICU of Showa University Hospital from November 2020 to August 2021. The participants were infants born at less than 32 weeks of gestation or with a birth weight of less than 1800 g. This study is an exploratory observational study; therefore, a statistical sample size calculation is not feasible. As a result, preterm infants hospitalized at our institution during a specific period were included in the study after obtaining consent from their parents. The zinc concentration in breast milk, serum zinc concentration in the infants, and the amount of zinc acetate supplementation were analyzed. Infants with severe congenital diseases or chromosomal abnormalities were excluded.

In our NICU, we routinely provide preterm and low-birthweight infants with parenteral nutrition (PN) and human milk fortification (HMF). PN was initiated at 2 g/kg/day of amino acids on the day of birth, and the dose was increased to a maximum of 3.5 g/kg/day. Fat emulsion started at 0.5 g/kg/day after one day of age, with the dose increased to a maximum of 2 g/kg/day. The water volume of PN and the doses of amino acids and fat gradually decreased as enteral nutrition increased. PN was terminated when enteral nutrition reached 120 mL/kg/day.

Mothers’ own milk (MOM) was given first priority for enteral nutrition and, when MOM was not available, human donor milk was used for infants weighing less than 1500 g at birth, while low-birthweight formula was used for infants weighing 1500–1800 g at birth. For infants in stable condition, enteral nutrition was initiated within 12 h of birth whenever possible, with the amount increased by 10–20 mL/kg/day, aiming for a final target of 140–160 mL/kg/day. Once enteral nutrition reached 50–100 mL/kg/day, HMF was introduced using powdered human milk fortifiers (HMS-1 or HMS-2; Morinaga Milk Industry, Tokyo, Japan).

As part of the nutritional management during NICU hospitalization, serum zinc levels were measured every 1–2 weeks from the day of birth, and oral administration of zinc acetate was started at 1–2 mg/kg/day based on the judgment of each attending physician, with the aim of maintaining serum zinc levels of 60–70 mcg/dL or higher. The dosage of zinc acetate was adjusted by the attending physician based on the serum zinc level, and the cutoff value for increasing or decreasing the dosage was not standardized.

After obtaining informed consent from the parents, the clinical background of the child, nutritional management, zinc concentration in breast milk, growth during hospitalization, other perinatal factors, and serum zinc concentration were prospectively followed. The following two groups were classified based on the maximum dose of zinc acetate administered during NICU hospitalization.

- High-dose group (HD group): maximum zinc acetate dose of 3 mg/kg/day or more
- Low-dose group (LD group): less than 3 mg/kg/day

Zinc concentration in the MOM was measured once a week. When defrosting frozen breast milk for daily feeding, 1 mL of excess breast milk was collected, immediately re-frozen at −20 °C, and then sent to the Shino-test Science Lab (Saitama, Japan) for measurement. If there was no excess MOM, zinc was not measured in the breast milk. The results of the zinc concentration measurements in breast milk were blinded to the attending physician, ensuring that they did not influence the adjustment of zinc dosage.

Z-scores for weight, length, and head circumference were calculated using the Japanese neonatal anthropometric charts reported by Itabashi et al. [14].

Statistical analyses were performed using the IBM SPSS Statistics software (version 27, IBM). For the comparison between the HD and LD groups, the Mann–Whitney U test was used for continuous variables, while Fisher’s exact test or Pearson’s chi-square test was used for categorical variables, and *p* < 0.05 was considered statistically significant in both cases.

This study was approved by the Showa University Research Ethics Review Board (approval no.: 3213). When conducting the study, parents were informed of the content of the study, and written consent was obtained.

## 3. Results

### 3.1. Patient Background

During the study period, 36 cases (20 boys and 16 girls) were included. The median gestational age was 30.9 weeks (range: 24.1–38.1 weeks), and the median birth weight was 1364 g (597–1757 g). All patients received PN from birth, and the median duration of PN was 8.5 days. The median age at which enteral nutrition reached 100 mL/kg/day was 7 days, and the median age at which HMF was initiated was 7 days. Participant characteristics are detailed in Table 1.

Complications during NICU hospitalization included respiratory distress syndrome (RDS) in 61.1% of the cases, patent ductus arteriosus requiring treatment in 33.3%, bronchopulmonary dysplasia (BPD) at 36 weeks of corrected age in 27.8%, and retinopathy of prematurity (ROP) requiring treatment in 5.6%. None of the infants had necrotizing enterocolitis (NEC).

Zinc acetate was administered to 32 of the 36 patients (88.9%), with a median age of 29.5 days (range: 13–69 days) at the start of administration. At the time of discharge, none of the patients were completely formula-fed, and approximately 40% were fully breastfed.

### 3.2. Zinc Concentration in MOM

Table 2 shows the change in zinc concentration in the MOM from 0 weeks (0–6 days after birth) to 8 weeks (56–62 days after birth). The zinc concentration in the MOM was highest immediately after delivery and then decreased over time, falling to approximately half at 4 weeks after birth and approximately a quarter at 8 weeks after birth.

### 3.3. Serum Zinc Concentration on the Day of Birth

**Figure 1:**
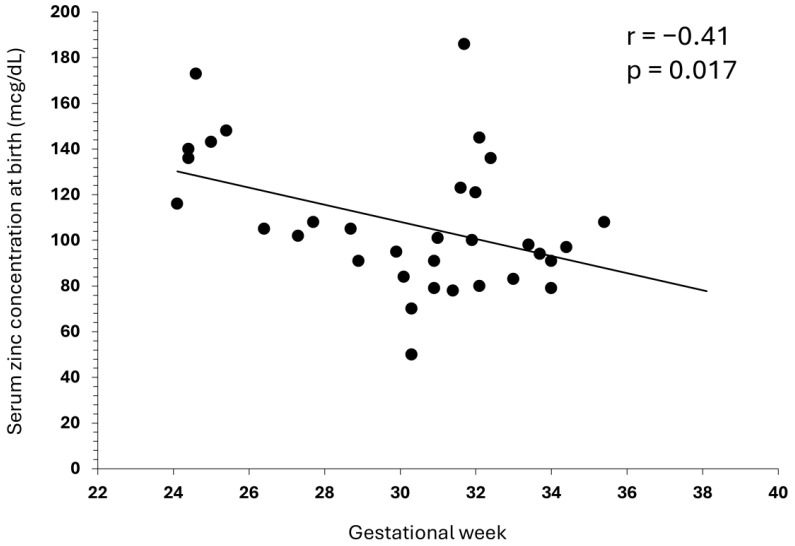
Correlation between gestational age and serum zinc concentration at birth. In the 33 cases where zinc was measured at birth, a weak negative correlation was observed between gestational age and serum zinc concentration at birth (r = −0.41, *p* = 0.017).

In the 33 cases where serum zinc concentration was measured on the day of birth, a weak negative correlation (r = −0.41, *p* = 0.017) was observed between serum zinc concentration at birth and gestational age (Figure 1). In three cases, the serum zinc concentration on the day of birth could not be measured because of insufficient blood sample volume.

### 3.4. Comparison Based on the Maximum Dose of Zinc Acetate Preparations

Based on the maximum dose of zinc acetate, the group that received 3 mg/kg/day or more was defined as the HD group (High-Dose group, *n* = 13), and the group that received less than 3 mg/kg/day was defined as the LD group (Low-Dose group, *n* = 23). Table 3 presents the clinical backgrounds of the two groups. In the HD group, there was a tendency toward shorter gestational age and lower birth weight, length, and head circumference; however, there were no statistically significant differences. There were also no significant differences between the two groups in terms of PN duration, the age at which enteral nutrition reached 100 mL/kg/day, or the age at which HMF was initiated. There were also no differences in body size around the expected date of delivery or the incidence of complications during hospitalization.

When comparing the zinc concentrations in the MOM of the HD and LD groups, the zinc concentration in the MOM of the HD group was significantly lower at 2, 4, and 5 weeks after birth, but the difference between the two groups disappeared after 6 weeks (Table 4). Regarding the serum zinc concentration for the infants, the HD group showed significantly higher levels only at 5–6 weeks of age (35–48 days old), but no significant differences were observed at other time points (Table 5). None of the children in either group exhibited clinical symptoms of zinc deficiency.

## 4. Discussion

In this study, we prospectively evaluated the relationship between the zinc concentration in the breast milk of mothers who had given birth to preterm infants and the zinc concentration in the infant serum. It is known that the zinc concentration in breast milk is highest in the colostrum immediately after delivery and then decreases over time [6,7]. A similar trend was observed in the present study.

Among the 36 preterm infants enrolled in this study, those who required more than 3 mg/kg/day of zinc tended to have shorter gestational ages and lower birth weights, although this difference was not statistically significant. A significant but weak negative correlation was observed between serum zinc concentration on the day of birth and gestational age, indicating that shorter gestational age is associated with higher serum zinc concentration. Although this study had a small sample size, which should be considered when interpreting the results, it has been reported that zinc is actively transported from the mother to the fetus, with accumulation in fetal tissues notably increasing from the third trimester onwards [3,4,5]. Therefore, while preterm infants may have high serum zinc concentrations immediately after birth, they possess low levels of zinc accumulation in their bodies.

Furthermore, the results of this study showed that the zinc concentration in MOM was low in preterm infants who required >3 mg/kg/day of zinc. However, in clinical practice, it is not realistic to measure the zinc concentration in breast milk over time. Therefore, it is essential to regularly monitor serum zinc concentrations and adjust the dosage for the nutritional management of preterm infants. In addition, there was no difference in serum zinc levels between the HD and LD groups in this study. This is believed to be due to appropriate supplementation and the monitoring of zinc levels over time.

There remains significant debate regarding the appropriate dose of zinc supplementation for preterm infants. In Japan, the *Clinical Practice Guidelines for Zinc Deficiency* recommend a daily dose of 1.0–1.5 mg/kg for infants and children; however, there is no standardized dose for preterm infants [11]. In contrast, Western nutritional guidelines recommend a daily dose of 1.4–2.5 mg/kg according to the AAP [12], while the ESPGHAN recommends a daily dose of 2–3 mg/kg [13]. However, due to low zinc bioavailability in preterm infants and high fecal excretion rates [3,15], there are reports suggesting that higher doses of 3–5 mg/kg/day or more may be necessary [16]. Since human milk fortifiers used in Japan do not contain zinc, it is assumed that the risk of zinc deficiency in preterm infants during the early stages of life is high. Therefore, it is likely that the required amount cannot be achieved with the same 1.0–1.5 mg/kg/day recommended for general infants.

Dermatitis and growth disorders are generally known symptoms of zinc deficiency; however, in recent years, it has been suggested that zinc deficiency may also affect the mortality and morbidity associated with complications specific to preterm infants, such as NEC, BPD, ROP, and neurodevelopmental outcomes after discharge from the NICU [17,18,19]. In this study, we evaluated only the short-term outcomes and found no significant differences in the growth or incidence of these complications during NICU hospitalization between the HD and LD groups. The incidence of NEC is low, and that of BPD and ROP is high in Japan [20]. In the future, large-scale prospective studies are needed to determine the impact of aggressive zinc supplementation on the risk of these complications.

This study had some limitations. This non-interventional observational study aimed to evaluate the relationship between zinc concentrations in MOM and serum zinc concentrations in infants in the context of current nutritional management. Therefore, the timing and dosage of zinc acetate administration were left to the discretion of the attending physician and were not standardized. In addition, the oral administration of zinc preparations can inhibit the absorption of copper and may cause copper deficiency [21]. Monitoring serum copper is desirable for evaluating the safety of zinc administration; however, serum copper was not measured in this study, which is one of its limitations. However, clinically, there were no symptoms suggesting copper deficiency, such as anemia that was unresponsive to iron preparations, or neutropenia.

Furthermore, the participants in this study included infants who were not exclusively breastfed. However, the results suggest that, even if a baby is not exclusively breastfed, there is a high risk of zinc deficiency if the zinc concentration in the MOM is low. Finally, this study was conducted at a single institution, and the number of subjects was limited. Therefore, a large-scale study using a standardized zinc administration protocol is required.

## 5. Conclusions

This study showed that there are individual differences in the zinc concentration in breast milk and that the MOM of preterm infants who require high-dose zinc supplementation has low zinc content. Ideally, it is desirable to use zinc-containing human milk fortifiers in Japan in the same manner as in other countries. However, at present, zinc is not added to human milk fortifiers; therefore, it is necessary to be fully aware of the high risk of zinc deficiency in preterm and low-birthweight infants in the early postnatal period.

In the nutritional management of preterm infants, it is important to regularly monitor serum zinc levels and provide zinc supplementation at appropriate times. In the future, there will be a need to standardize the management guidelines for preterm infants, including the appropriate dosage and timing of zinc administration.

## Figures and Tables

**Table 1:** Participant characteristics (*n* = 36).

Characteristics	Number (%) or Median (Range)
Clinical backgrounds at birth	
Gestational age (weeks)	30.9 (24.1–38.1)
Male	20 (55.6)
BW at birth (grams)	1364 (597, 1757)
BW at birth (z-score)	−0.78 (−4.01, 1.10)
BW < 10% tile at birth	13 (36.1)
Length at birth (cm)	38.3 (30.0, 45.0)
Length at birth (z-score)	−0.65 (−2.41, 2.84)
Length < 10% tile at birth	12 (33.3)
HC at birth (cm)	27.9 (21.0, 31.0)
HC at birth (z-score)	−0.31 (−2.86, 1.29)
HC < 10% tile at birth	5 (13.9)
Primiparas	23 (63.9)
Multiple birth	11 (30.6)
Apgar score at 1 min	6 (1–9)
Apgar score at 5 min	8 (4–10)
Caesarean section	30 (83.3)
Nutritional care during NICU hospitalization	
Age at which weaning from parenteral nutrition (days)	8.5 (4, 24)
Age at which enteral feeding reached 100 mL/kg/day (days)	7 (5, 19)
Age at which human milk fortification initiated (days)	7 (5, 24)
Percentage of breast milk at NICU discharge	
0%	0 (0)
1–49%	9 (25.0)
50–99%	12 (33.3)
100%	15 (41.7)
Complications during NICU hospitalization	
RDS	22 (61.1)
PDA	12 (33.3)
Early onset sepsis	2 (5.6)
Late onset sepsis	2 (5.6)
NEC	0 (0)
BPD at 36 weeks PMA	10 (27.8)
ROP with treatment	2 (5.6)
Severe IVH (≥grade3)	3 (8.3)
Cystic PVL	1 (2.8)
Anthropometric measurements at 40 weeks PMA	
BW at 40 weeks PMA or NICU discharge (grams)	2590 (1590, 3988)
BW at 40 weeks PMA or NICU discharge (z-score)	−1.20 (−5.11, 2.43)
BW < 10% tile at 40 weeks PMA or NICU discharge	17 (47.2)
Length at 40 weeks PMA or NICU discharge (cm)	45.5 (41.0, 52.5)
Length at 40 weeks PMA or NICU discharge (z-score)	−1.79 (−4.09, −0.98)
Length < 10% tile at 40 weeks PMA or NICU discharge	9 (69.2)
HC at 40 weeks PMA or NICU discharge (cm)	33.5 (30.7, 36.5)
HC at 40 weeks PMA or NICU discharge (z-score)	0.08 (−2.03, 2.53)
HC < 10% tile at 40 weeks PMA or NICU discharge	2 (5.6)

BW, body weight; HC, head circumference; NICU, neonatal intensive care unit; RDS, respiratory distress syndrome; PDA, patent ductus arteriosus; NEC, necrotizing enterocolitis; BPD, bronchopulmonary dysplasia; PMA, postmenstrual age; ROP, retinopathy of prematurity; IVH, intraventricular hemorrhage; PVL, periventricular leukomalacia.

**Table 2:** Changes in zinc concentration in breast milk.

Postpartum Week	Number of Samples	Zinc Concentration (mcg/dL) Median (Range)
Week 0 (day 0–6)	15	466 (267, 1061)
Week 1 (day 7–13)	22	443 (297, 952)
Week 2 (day 14–20)	28	324 (238, 734)
Week 3 (day 21–27)	28	295.5 (175, 628)
Week 4 (day 28–34)	27	253 (121, 666)
Week 5 (day 35–41)	23	185 (124, 499)
Week 6 (day 42–48)	18	141 (101, 429)
Week 7 (day 49–55)	11	138 (87, 254)
Week 8 (day 56–62)	10	120.5 (88, 370)

**Table 3:** Comparison of characteristics between HD and LD groups.

Characteristics	Number (%) or Median (Range)		*p* Value
	HD Group (*n* = 13)	LD Group (*n* = 23)	
Clinical backgrounds at birth			
Gestational age (weeks)	27.7 (24.4, 34.0)	31.7 (24.1, 38.1)	0.084
Male	6 (46.2)	14 (60.9)	0.393
BW at birth (grams)	1089 (597, 1653)	1409 (608, 1757)	0.084
BW at birth (z-score)	−0.39 (−2.70, 0.48)	−0.95 (−4.01, 1.10)	0.172
BW < 10% tile at birth	2 (15.4)	11 (47.8)	0.054
Length at birth (cm)	38.0 (30.0, 40.0)	39.0 (30.0, 45.0)	0.082
Length at birth (z-score)	−0.37 (−1.99, 2.84)	−0.78 (−2.41, 1.76)	0.587
Length < 10% tile at birth	4 (30.8)	8 (34.8)	0.553
HC at birth (cm)	27.0 (21.0, 29.4)	28.3 (21.3, 31)	0.058
HC at birth (z-score)	−0.25 (−1.36, 1.29)	−0.37 (−2.86, 1.07)	0.479
HC < 10% tile at birth	1 (7.7)	4 (17.4)	0.395
Primiparas	11 (84.6)	12 (52.2)	0.054
Multiple birth	6 (46.2)	5 (21.7)	0.126
Apgar score at 1 min	6 (1, 8)	7 (1, 9)	0.867
Apgar score at 5 min	8 (4, 9)	8 (4, 10)	0.958
Caesarean section	10 (76.9)	20 (87.0)	0.438
Nutritional care during NICU hospitalization			
Age at which weaning from parenteral nutrition (days)	8 (4, 17)	8 (4, 10)	0.343
Age at which enteral feeding reached 100 mL/kg/day (days)	6 (4, 17)	7 (5, 19)	0.534
Age at which human milk fortification initiated	7 (5, 19)	7 (5, 24)	0.343
Percentage of breast milk at NICU discharge (days)			
0%	0 (0)	0 (0)	0.603
1–49%	4 (30.8)	5 (21.7)	
50–99%	3 (23.1)	9 (39.1)	
100%	6 (46.2)	9 (39.1)	
Complications during NICU hospitalization			
RDS treated with surfactant	9 (69.2)	13 (56.5)	0.452
PDA	5 (38.5)	7 (30.4)	0.447
Early onset sepsis	0 (0)	2 (8.7)	
Late onset sepsis	1 (7.7)	1 (4.3)	0.402
NEC	0 (0)	0 (0)	0.598
BPD at 36 weeks PMA	6 (46.2)	4 (17.4)	0.073
ROP with treatment	2 (15.4)	0 (0)	0.124
Severe IVH (≥grade3)	2 (15.4)	1 (4.3)	0.291
Cystic PVL	0 (0)	1 (4.3)	0.639
Anthropometric measurements at 40 weeks PMA			
BW at 40 weeks PMA or NICU discharge (grams)	2602 (2387, 2928)	2530 (1590, 3988)	0.365
BW at 40 weeks PMA or NICU discharge (z-score)	−0.89 (−2.17, 0.14)	−1.42 (−5.11, 2.43)	0.229
BW < 10% tile at 40 weeks PMA or NICU discharge	4 (30.8)	13 (56.5)	0.137
Length at 40 weeks PMA or NICU discharge (cm)	44.5 (41.5, 47.5)	46.0 (41.0, 52.5)	0.170
Length at 40 weeks PMA or NICU discharge (z-score)	−1.89 (−4.09, -0.98)	−1.72 (−3.37, 2.06)	0.143
Length < 10% tile at 40 weeks PMA or NICU discharge	9 (69.2)	14 (60.9)	0.448
HC at 40 weeks PMA or NICU discharge (cm)	33.5 (32.0, 35.2)	33.2 (30.7, 36.5)	0.408
HC at 40 weeks PMA or NICU discharge (z-score)	0.08 (-0.68, 1.38)	−0.06 (−2.03, 2.53)	0.383
HC < 10% tile at 40 weeks PMA or NICU discharge	0 (0)	2 (8.7)	0.402

BW, body weight; HC, head circumference; NICU, neonatal intensive care unit; RDS, respiratory distress syndrome; PDA, patent ductus arteriosus; NEC, necrotizing enterocolitis; BPD, bronchopulmonary dysplasia; PMA, postmenstrual age; ROP, retinopathy of prematurity; IVH, intraventricular hemorrhage; PVL, periventricular leukomalacia.

**Table 4:** Comparison of zinc concentration in breast milk between HD and LD groups.

Postpartum Week	HD Group (*n* = 13)		LD Group (*n* = 23)		*p* Value
	Number of Samples	Zinc Concentration (mcg/dL)	Number of Samples	Zinc Concentration (mcg/dL)	
Week 0 (day 0–6)	4	466 (399, 886)	11	471 (267, 1061)	0.896
Week 1 (day 7–13)	9	385 (297, 765)	13	484 (333, 952)	0.242
Week 2 (day 14–20)	12	299.5 (238, 432)	16	410.5 (262, 734)	0.020
Week 3 (day 21–27)	10	271.5 (175, 429)	18	305.5 (192, 628)	0.230
Week 4 (day 28–34)	9	211 (121, 253)	18	330.5 (153, 666)	0.002
Week 5 (day 35–41)	7	179 (124, 185)	16	261.5 (155, 499)	0.041
Week 6 (day 42–48)	8	134.5 (101, 165)	10	200 (114, 429)	0.154
Week 7 (day 49–55)	6	148 (87, 254)	5	138 (119, 187)	0.713
Week 8 (day 56–62)	5	111 (88, 199)	5	205 (109, 370)	0.172

Zinc concentration is shown as the median (range).

**Table 5:** Comparison of serum zinc levels between HD and LD groups.

Weeks After Birth	HD Group (*n* = 13)		LD Group (*n* = 23)		*p* Value
	Number of Samples	Serum Zinc Level (mcg/dL)	Number of Samples	Serum Zinc Level (mcg/dL)	
Day at birth (day 0)	12	106.5 (79, 173)	21	97 (50, 186)	0.231
Week 1–2 (day 7–20)	4	73.5 (53, 100)	14	81 (56, 114)	0.489
Week 3–4 (day 21–34)	8	59.5 (46, 118)	18	65.5 (45, 91)	0.956
Week 5–6 (day 35–48)	7	83 (61, 140)	15	60 (50, 80)	0.002
Week 7–8 (day 49–62)	7	70 (43, 130)	12	68 (55, 149)	0.735

## Data Availability

The data presented in this study are available on request from the corresponding author (T.M.) due to privacy.

## Abbreviations

The following abbreviations are used in this manuscript:

AAP, American Academy of Pediatrics; EAN, Early aggressive nutrition; ESPGHAN, European Society for Pediatric Gastroenterology, Hepatology and Nutrition; HD, High-dose; HMF, Human milk fortification; LD, Low-dose; MOM, Mothers’ own milk; PN, Parenteral nutrition; ROP, Retinopathy of prematurity; BW, body weight; HC, head circumference; NICU, neonatal intensive care unit; PDA, patent ductus arteriosus; NEC, necrotizing enterocolitis; BPD, bronchopulmonary dysplasia; PMA, postmenstrual age; IVH, intraventricular hemorrhage; PVL, periventricular leukomalacia.
